# ASPP2 Coordinates ERS-Mediated Autophagy and Apoptosis Through mTORC1 Pathway in Hepatocyte Injury Induced by TNF-α

**DOI:** 10.3389/fphar.2022.865389

**Published:** 2022-03-28

**Authors:** Jia Yao, Hui Yang, Han Wang, Honglin Shi, Yan Jiao, Ying Zhang, Dexi Chen, Hongbo Shi

**Affiliations:** ^1^ Gastroenterology Department, General Surgery Department and Gastroenterology Department, ShanxiBethuneHospital, Shanxi Academy of Medical Sciences Tongji Shanxi Hospital, Third Hospital of Shanxi Medical University, Taiyuan, China; ^2^ Beijing Institute of Hepatology, Beijing Youan Hospital, Capital Medical University, Beijing, China; ^3^ Department of Nephrology, Army Medical Center, Army Medical University, Chongqing, China; ^4^ Beijing Engineering Research Center for Precision Medicine and Transformation of Hepatitis and Liver Cancer, Beijing, China

**Keywords:** hepatocyte injury, ASPP2, mTORC1, ERS, apoptosis, autophagy

## Abstract

**Background:** Though ASPP2 plays an important role in regulating cell apoptosis and autophagy in case of liver injury, there remains a lack of clarity on the molecular mechanism of ASPP2 regulating autophagy and apoptosis.

**Methods:** A hepatocyte injury model was constructed using HL7702 cell line and TNF-α. The cells were treated by ASPP2 overexpression adenovirus or short hairpin RNA lentivirus and endoplasmic reticulum stress (ERS) or the mammalian target of rapamycin (mTOR) inhibitor or agonist, respectively. The autophagy was detected by means of western blot and Green fluorescent protein-labeled- Microtubule-associated protein light chain 3 (GFP-LC3) plasmid transfection, while the apoptosis was detected through western blot, flow cytometry and TUNEL assay. Besides, the proteins related to ERS and mTOR were detected by western blot.

**Results:** The low level of ASPP2 expression was accompanied by high-level autophagy and low-level apoptosis and vice versa in case of hepatocyte injury induce by TNF-α. By upregulating the proteins related to mTORC1 and ERS, ASPP2 induced apoptosis but inhibited autophagy. However, the effect of ASPP2 on autophagy and apoptosis can be reversed by the use of mTORC1 and ERS interfering agent, which indicates that ASPP2 regulated autophagy and apoptosis through mTORC1and ERS pathway. ERS treatment made no difference to the expression of ASPP2 and mTOR-related proteins, which suggests the possibility that the regulation of ERS on apoptosis and autophagy could occur in the downstream of ASPP2 and mTOR.

**Conclusion:** ASPP2 could inhibit autophagy and induce apoptosis through mTORC1-ERS pathway in case of the hepatocyte injury induce by TNF-α. The role of ASPP2-mTORC1-ERS axis was verified in hepatocyte injury, which suggests the possibility that ASPP2 is an important regulatory molecule for the survival and death of hepatocyte.

## Introduction

Liver inflammation and injury can be caused by virus, high-fat diet, alcohol, drugs and other risk factors (Seitz et al., 2018). In addition to the direct impact on hepatocytes, these risk factors will end up activating liver kupffer cells, which leads to the release of tumor necrosis factor α (TNF-α), interleukin 6 (IL-6) and other inflammatory factors, thus causing hepatocyte apoptosis and necrosis. This is referred to as the theory of hepatocyte immune damage or second hit (Osna et al., 2017). Therefore, to study the hepatocyte injury induced by TNF-α plays an important role in guiding the treatment of liver inflammatory injury caused by many factors.

As a member of ASPP family, apoptosis stimulating protein 2 of p53 (ASPP2) includes ASPP1, ASPP2, and iASPP. Among them, ASPP2 was initially considered to be a p53 binding protein, and it was screened from yeast two hybrid system with p53 as bait. ASPP2 plays a significant role in regulating cell apoptosis, growth and differentiation (Vives et al., 2006; Turnquist et al., 2014; Li et al., 2015). As discovered by our research group, in the acute hepatitis mouse model induced by CCl_4_, both liver inflammatory damage and hepatocyte apoptosis were significantly mitigated for ASPP2 heterozygous deletion (ASPP2^+/−^) mice. By inhibiting autophagy, ASPP2 promoted hepatocyte apoptosis and liver inflammation (Xu et al., 2019). However, there is still no clarity about the molecular mechanism of ASPP2 regulating autophagy and apoptosis, which makes it necessary to further explore the role of ASPP2 in the correlation between autophagy and apoptosis.

As a typical signaling molecular pathway, the mammalian target of rapamycin (mTOR) is capable to receive signals from growth factors, hormones and cytokines. Besides, it is closely related to the regulation of energy metabolism and stress response (Hua et al., 2019). As another variety of regulatory molecules in the upstream of autophagy, mTORC1 can suppress the initial formation of autophagic vesicles by inhibiting ATG13 (Odle et al., 2020). In addition to inhibiting autophagy, mTORC1 also regulates the growth and proliferation of cells through two different types of downstream molecule, namely, P70S6K and 4E-BP1 (Rindom et al., 2020). In our previous study, it was demonstrated that ASPP2 can regulate liver regeneration and hepatocyte proliferation through mTORC1 pathway (Shi et al., 2018), despite the lack of clarity on whether ASPP2 could regulate autophagy and apoptosis through mTORC1 pathway.

In this study, it was discovered that ASPP2 promoted endoplasmic reticulum stress (ERS) mediated apoptosis but inhibited autophagy by upregulating mTORC1 in case of hepatocyte injury induced by TNF-α, suggesting that ASPP2 may serve as an essential regulatory molecule in cell survival and death.

## Materials and Methods

### Materials

HL7702 cell lines were obtained from the American Type Culture Collection (ATCC). 1640 medium, fetal bovine serum, trypsin, and streptomycin were purchased from Gibco. ASPP2 adenovirus (Adv-ASPP2), control adenovirus (Adv-control), ASPP2 short hairpin RNA lentivirus (LV-ASPP2shRNA) and control short hairpin RNA lentivirus (LV-controlshRNA) were purchased from Beijing He Sheng Gene Technology Company. Green fluorescent protein-labeled- autophagy marker light chain 3 (GFP-LC3) plasmid was provided by Beijing Biomed Gene Technology Company. Endoplasmic reticulum stress inducer Tunicamycin (TM), endoplasmic reticulum stress inhibitor (4-PBA), mTOR inhibitor rapamycin and mTOR agonist MHY1485 were sourced from Sigma Company.

### Cell Culture

HL7702 was cultured in 1640 medium with 10% fetal calf serum and 1% penicillin streptomycin for incubation in a 37°C, 5% CO_2_ incubator. Then, these cells were treated with 25, 50, and 100 (ng/ml) TNF-α for 12, 24, and 48 h, respectively. In contrast, the control group was not treated with TNF-α. The cells were first transfected by Adv-ASPP2 or Adv-control (5 nM), then pretreated by 4-PBA (2 mM) or rapamycin (1 μmmol/L) for 2 h and finally induced by TNF-α (25 ng/ml) for 24 h. Those stable cell lines were first prepared using LV-ASPP2shRNA or LV-controlshRNA, then treated with TM (20 ng/ml) or MHYI485 (5 umo/L) and finally induced by TNF-α (25 ng/ml) for 24 h.

### GFP-LC3 Plasmid Transfection

According to the protocol provided by Roche, the GFP-LC3 plasmid was transfected into cells using the X-tremeGENE HP DNA Transfection Reagent transfection method. The nuclei were visualized by staining with 4,6-dimidyl-2phenylindole (DAPI), and the green spots of cells were manually counted in five randomly selected areas under a Nikon Eclipse E800 fluorescence microscope.

### Western Blot

These cells were lysed on ice for 30 min with the assistance of RIPA buffer containing protease inhibitor. Protein was detected by SDS-PAGE and then transferred to a polyvinylidene fluoride (PVDF) membrane. The membrane was incubated using the appropriate primary antibody (ASPP2, LC3B from Sigma and caspase3, cleaved caspase3, Glucose-Regulated Protein 78 (GRP78), Chop, p62 (sequestosome 1), phospho-mTOR, phospho-S6 (Ser235/236), phospho-p70S6K (Thr389) and β-actin antibody from cell signaling technology) at 4°C. After being washed for three times, the membrane was incubated with the corresponding secondary antibody (cell signaling technology) for 1 h at room temperature. Finally, the membrane was photographed using a gel imaging system. Moreover, optical density analysis was conducted using ImageJ software, with the relative levels of proteins in each group normalized to a loading control.

### Flow Cytometry Detection

Annexin V-Phycoerythrin (PE) and 7-Amino-Actinomycin (7-AAD) double staining method (BD Bioscience, Franklin Lakes, NJ, United States) was adopted to detect apoptosis. HL-7702 cells (Human normal liver cell line-7702) were cultured in a 6-well plate at a density of 1 × 10^6^ cells per well. After treatment, the cells were suspended in binding buffer, and then stained with PE and 7-AAD reagents for 15 min in the dark. The samples were analyzed using the BD FACS Calibur flow cytometer, and the data was analyzed using FlowJo software.

### TUNEL Assay

According to the instructions provided for the apoptosis detection kit (KeyGEN BioTECH, Nanjing, China), terminal deoxynucleotidyl transferasemediated deoxyuridine triphosphate nick end labeling (TUNEL) was performed to detect the apoptotic cells. Climbing on the slices, the cells were fixed in 4% paraformaldehyde for 20 min and permeabilized with 0.5% triton-X 100 solution. After being washed with PBS buffer for three times, the cells were added with fluorescence labeled TUNEL mixture, and the nuclei were counterstained by DAPI. Afterwards, the cells were observed under Nikon Eclipse E800 fluorescence microscope, and the apoptotic cells were counted using ImageJ software.

### Statistical Analysis

With all data were expressed as mean ± SD, *t* test was conducted on two sets of data using GraphPad Prism 7.0 software. Followed by post hoc LSD, one-way analysis of variance (ANOVA) was carried out to draw comparison between more than two groups, with *p* < 0.05 treated as statistically significant.

## Results

### TNF-α Activates Autophagy in Low Dose and Induces Apoptosis in High Dose, but Stimulates ASPP2 Expression During the Entire Experimental Course

**FIGURE 1:**
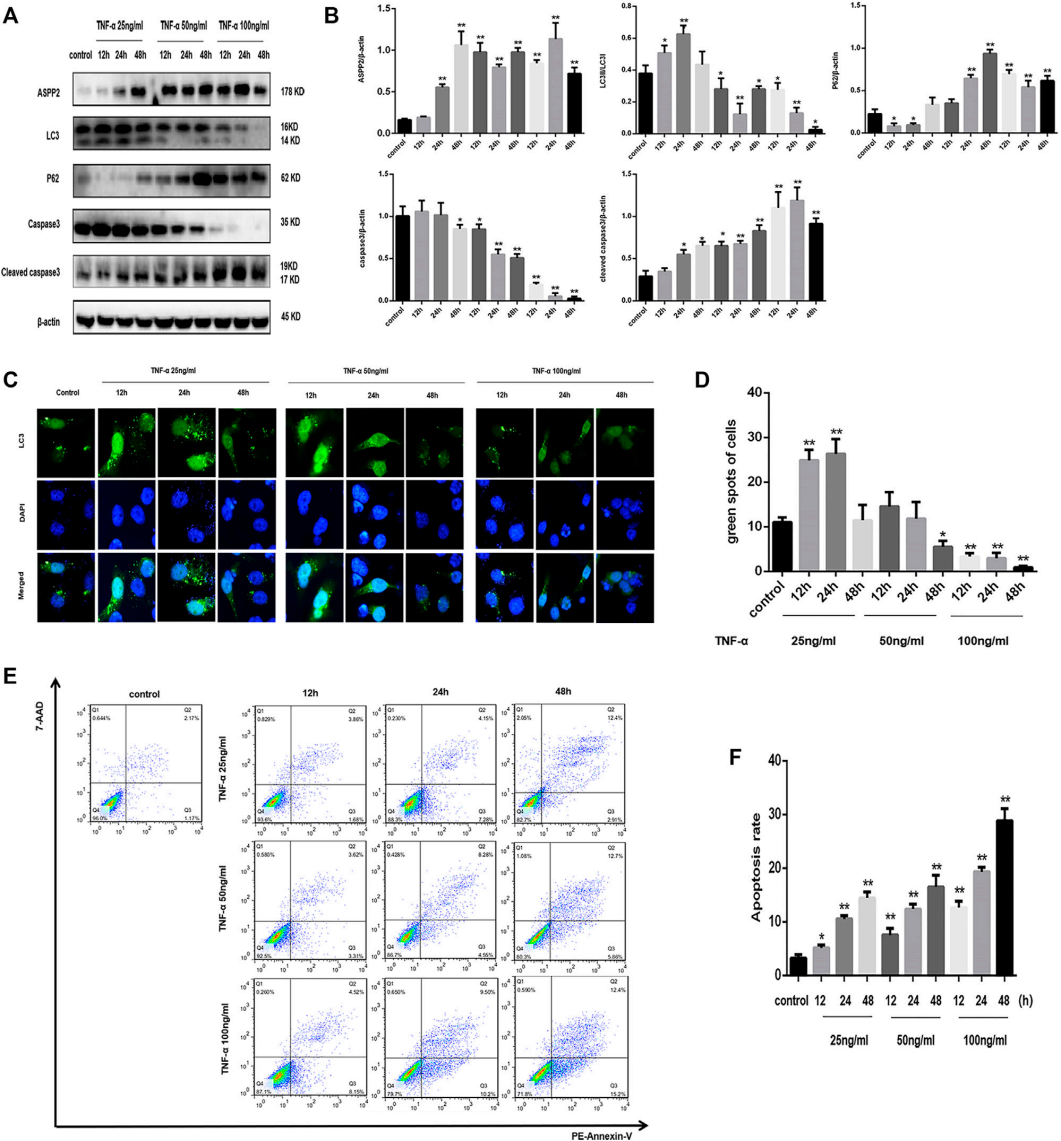
TNF-α activated autophagy in low dose and induced apoptosis in high dose, but triggered ASPP2 expression throughout the course of experiment. HL7702 cells were treated with 25, 50, and 100 (ng/ml) TNF-α for 12, 24, and 48 h, respectively. In contrast, the control group was not treated with TNF-α. **(A,B)** Representative western blotting analysis of protein expression in HL7702 cells after TNF-α treatment. Quantifications were normalized to β-actin and expressed as relative density. **(C,D)** A GFP-LC3 plasmid was transfected into cells and the representative fluorescence image of cells was presented. The green spots of cells were manually quantified in five randomly selected areas. **(E,F)** Representative apoptosis images were showed with PE and 7-AAD reagents by flow cytometry. The apoptosis rate was calculated using the proportion of early and late apoptotic cells in total cells. *p < 0.05; **p < 0.01.

It is widely known that ASPP2 is capable to regulate autophagy and apoptosis, but there is a lack of clarity about the molecular mechanism of ASPP2 in the interaction between autophagy and apoptosis. It was found out that ASPP2 expression was increased gradually with the low dose (25 ng/ml) of TNF-α, while ASPP2 maintained a high expression level given the medium and high dose (50 and 100 ng/ml) of TNF-α in 7702 cell line (Figures 1A,B). In addition, the conversion of LC3I into LC3II and the degradation of p62 were enhanced in 7702 cell line with the low dose TNF-α stimulation, which indicates the activation of high-level autophagy or autophagy (Figures 1A,B). Besides, the green fluorescent spots in 7702 cell line also suggests the formation of autophagosome, which is regarded as the marker of autophagy activation (Figures 1C,D). The medium or high dose TNF-α stimulation induced low-level autophagy or inhibited autophagy (Figures 1A,B). By contrast, the level of caspase3 expression was slightly reduced and that of cleaved caspase3 expression reached a slightly higher level than control in 7702 cell line with a low dose TNF-α stimulation, which indicates low-level apoptosis (Figures 1A,B). As revealed by flow cytometry, the amount of apoptotic cells increased slightly in 7702 cell line with given low-dose TNF-α stimulation. A high-dose TNF-α stimulation induced high-level apoptosis (Figures 1E,F). To sum up, the low level of ASPP2 expression was coupled with high-level autophagy and low-level apoptosis and vice versa in case of hepatocyte injury induce by TNF-α, which indicates that ASPP2 could play an important role in regulating autophagy and apoptosis.

### ASPP2 Upregulates mTORC1 and Inhibits Autophagy, but Promotes Apoptosis in Hepatocyte Injury Induce by TNF-α

**FIGURE 2:**
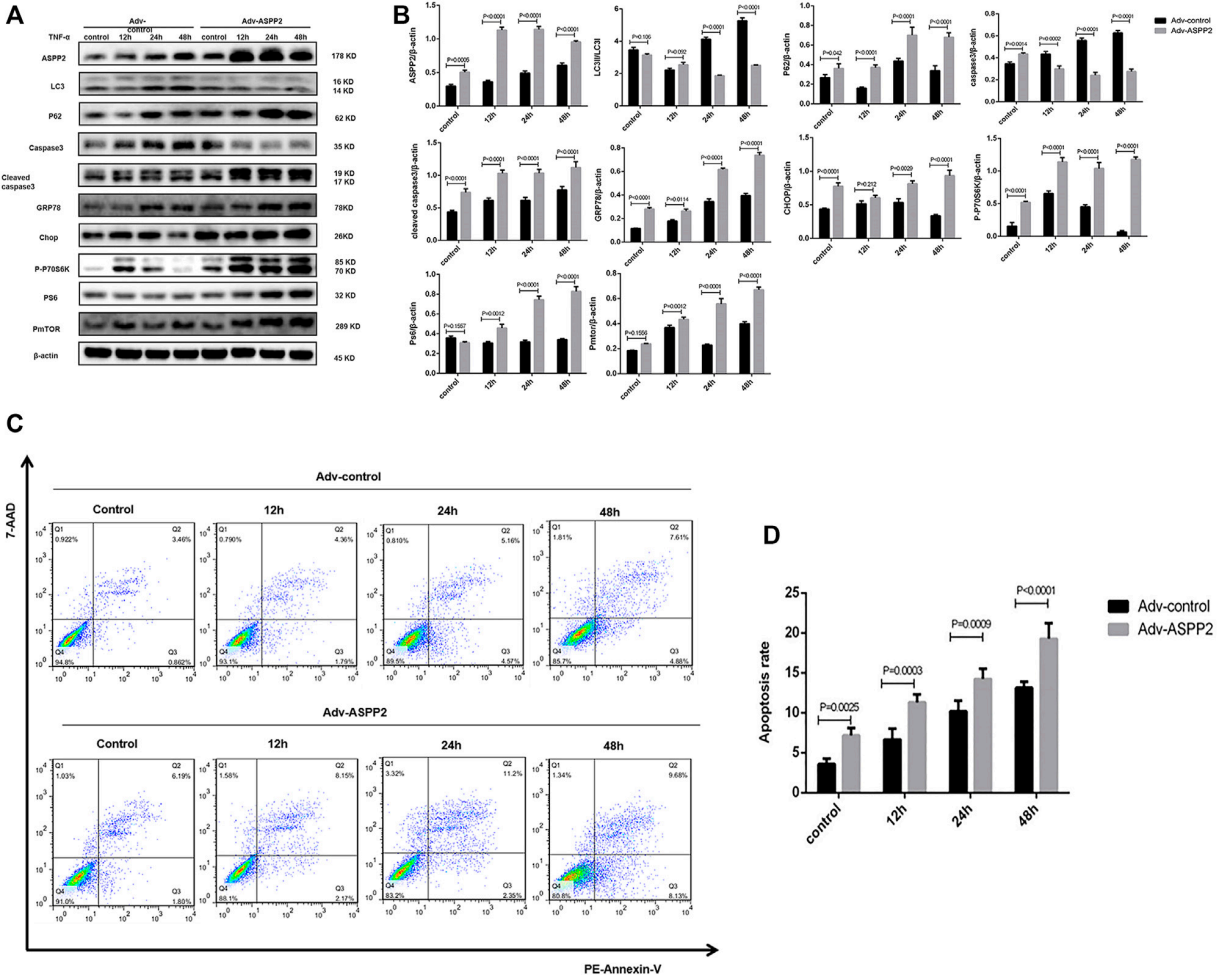
ASPP2 upregulated mTORC1 and inhibited autophagy, but promoted apoptosis in case of hepatocyte injury induce by TNF-α. HL7702 cells were transfected by Adv-ASPP2 or Adv-control and then induced by TNF-α (25 ng/ml) for 12, 24, and 48 h, respectively. **(A,B)** Representative western blotting analysis was conducted of protein expression in HL7702 cells after treatment. Quantifications were normalized to β-actin and expressed as relative density. **(C,D)** Representative apoptosis images were presented with PE and 7-AAD reagents by flow cytometry. The apoptosis rate was calculated using the proportion of early and late apoptotic cells in total cells.

**FIGURE 3:**
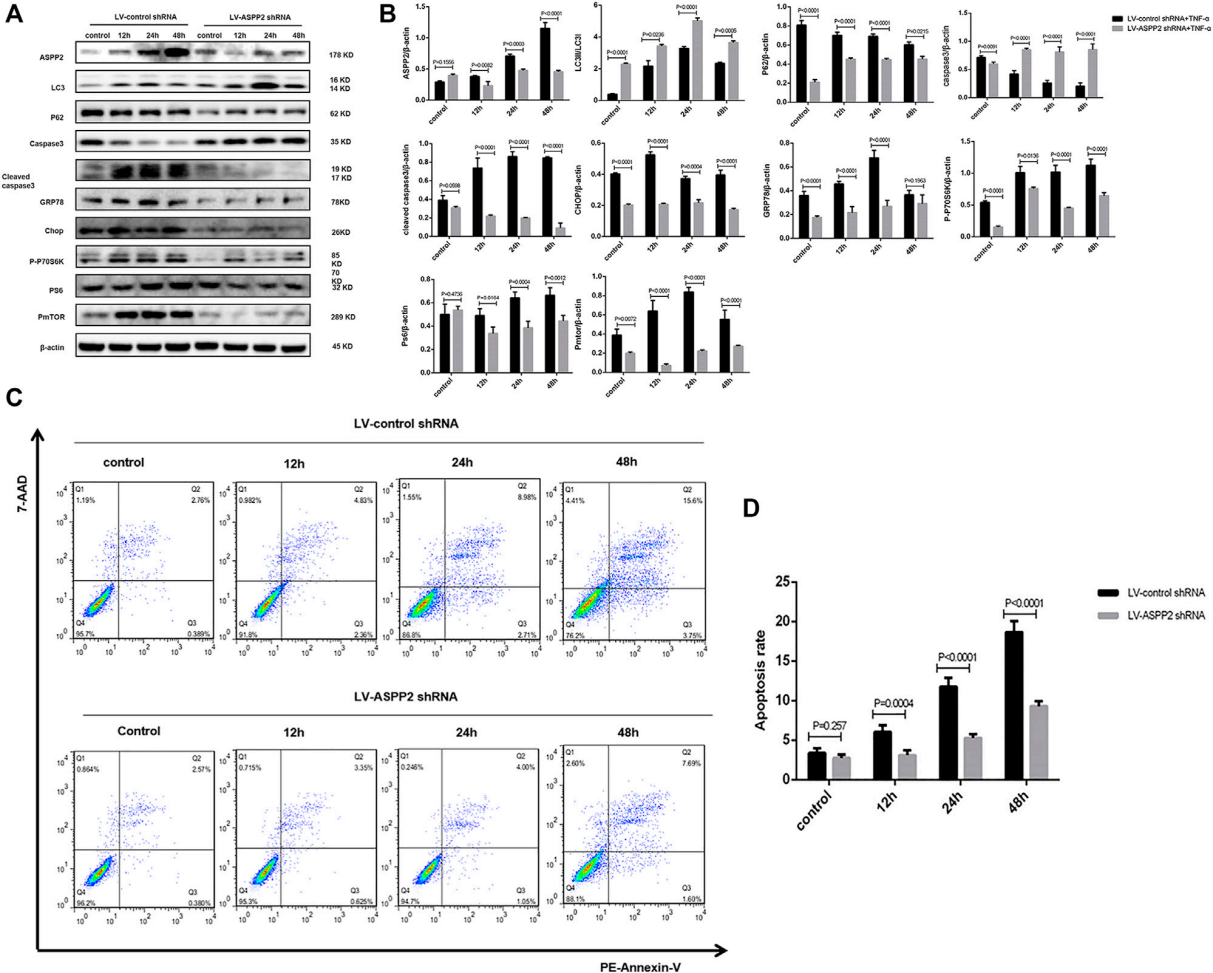
ASPP2 inhibition downregulated mTORC1 and enhanced autophagy, but inhibited apoptosis in case of hepatocyte injury induce by TNF-α. HL7702 cells were transfected by LV-ASPP2shRNA or LV-control shRNA and then induced by TNF-α (25 ng/ml) for 12, 24, and 48 h, respectively. **(A,B)** Representative western blotting analysis was conducted of protein expression in HL7702 cells after treatment. Quantifications were normalized to β-actin and expressed as relative density. **(C,D)** Representative apoptosis images were presented with PE and 7-AAD reagents by flow cytometry. The apoptosis rate was calculated using the proportion of early and late apoptotic cells in total cells.

ASPP2 inhibited autophagy, as evidenced by the reduced conversion of LC3I to LC3II and increased accumulation of p62 in those cells treated with ASPP2 adenovirus compared to control (Figures 2A,B). ASPP2 promoted autophagy, as reflected in the suppressed caspase3 expression and enhancement of cleaved caspase3 expression in those cells treated with ASPP2 adenovirus compared to control (Figures 2A,B). According to flow cytometry, there were more apoptotic cells among the cells treated with ASPP2 adenovirus than in control (Figures 2C,D). Besides, ASPP2 upregulated the mTORC1 and endoplasmic reticulum stress pathway, as suggested by the increased expression of phosphorylated P70S6K (P-P70S6K), phosphorylated S6 (PS6), phosphorylated mTOR (PmTOR), Chop and GRP78 in those cells treated with ASPP2 adenovirus compared to control (Figures 2A,B). In contrast, the ASPP2 shRNA treatment in cells enhanced autophagy, ameliorated apoptosis, but downregulated mTORC1 and endoplasmic reticulum stress (ERS) pathway (Figure 3). In summary, ASPP2 regulated autophagy and apoptosis, which is possibly associated with mTORC1 and ERS pathway.

### ERS Promotes ASPP2 Mediated-Apoptosis, but Suppresses Autophagic Flux in Hepatocyte Injury Induce by TNF-α

**FIGURE 4:**
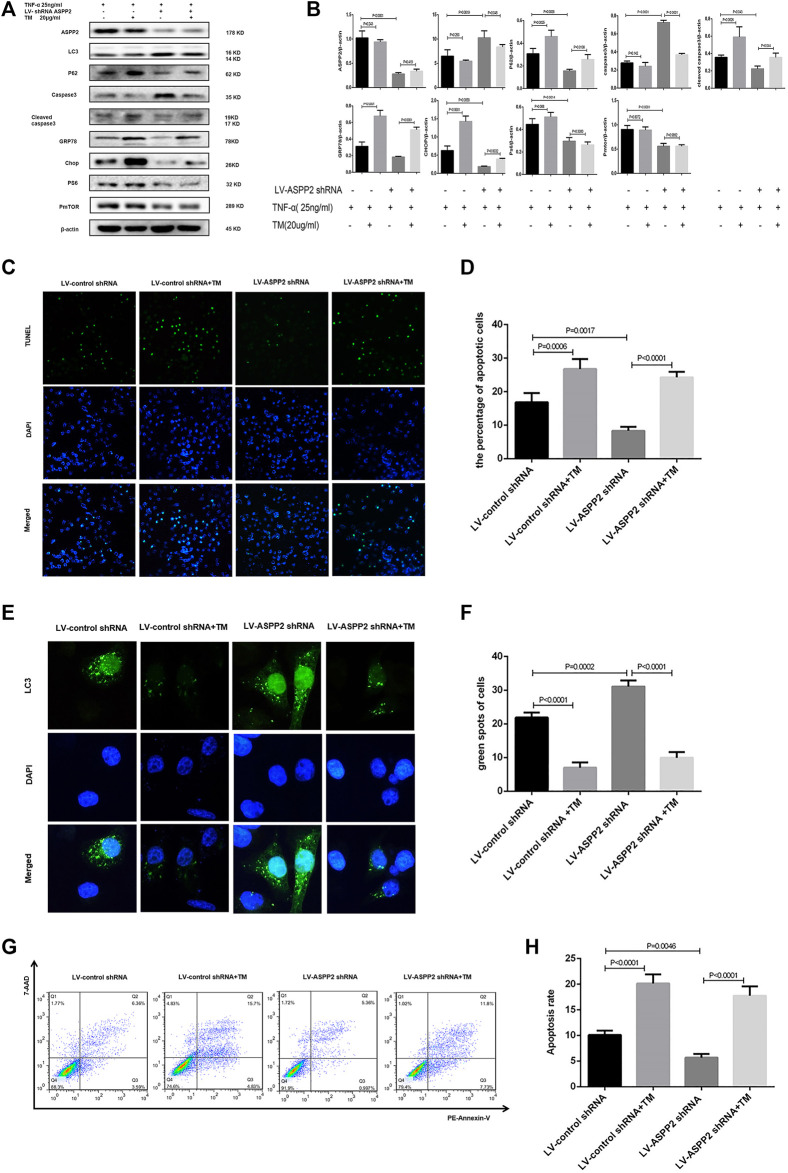
ERS promoted ASPP2 mediated-apoptosis, but suppressed autophagic flux in case of hepatocyte injury induce by TNF-α. HL7702 cells were integrated into stable cell lines using LV-ASPP2shRNA or LV-control shRNA. The stable cell lines were treated with TM (20 ng/ml) and then induced by TNF-α (25 ng/ml) for 24 h. **(A,B)** Representative western blotting analysis was conducted of protein expression in HL7702 cells after treatment. Quantifications were normalized to β-actin and expressed as relative density. **(C,D)** Representative apoptosis images were presented by TUNEL assay. The number of apoptotic cells were counted by ImageJ software. **(E,F)** A GFP-LC3 plasmid was transfected into cells and the representative fluorescence image of cells was showed. The green spots of cells were manually quantified in five randomly selected areas. **(G,H)** Representative apoptosis images were showed with PE and 7-AAD reagents by flow cytometry. The apoptosis rate was calculated using the proportion of early and late apoptotic cells in total cells.

**FIGURE 5:**
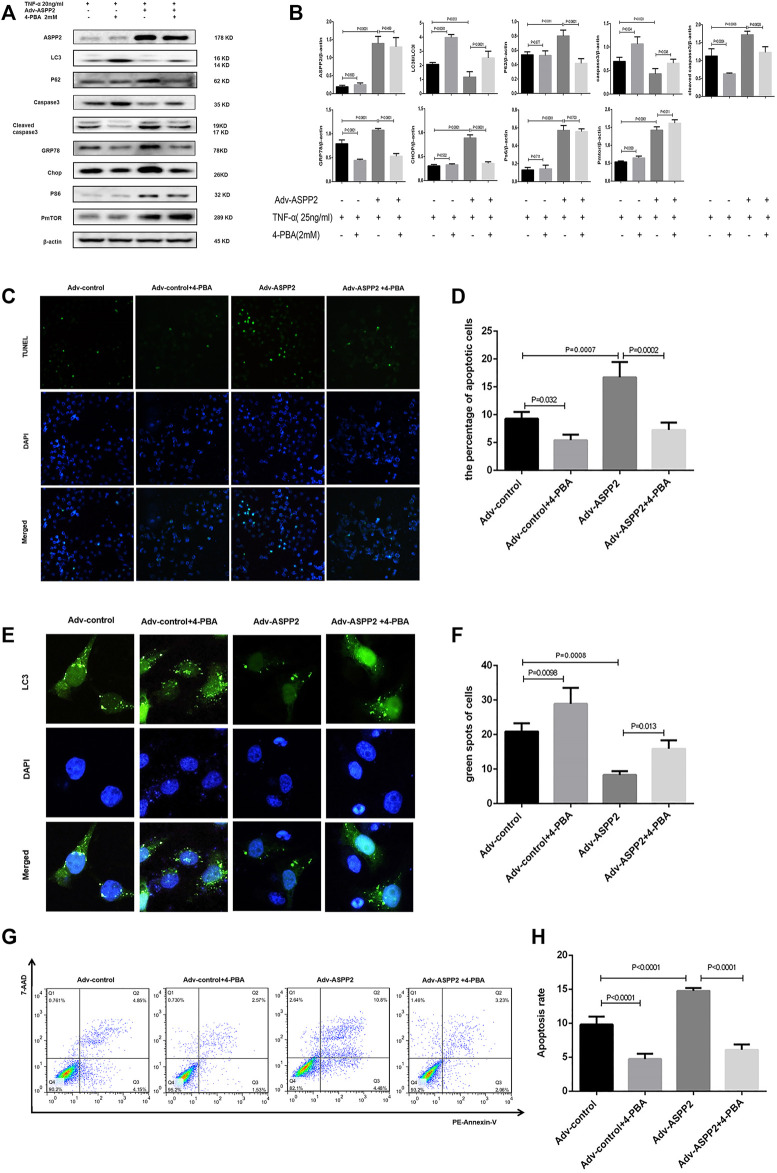
ERS inhibition suppressed ASPP2 mediated-apoptosis, but enhanced autophagic flux in case of hepatocyte injury induce by TNF-α. HL7702 cells were transfected by Adv-ASPP2 or Adv-control (5 nM), then pretreated by 4-PBA (2 mM) for 2 h and finally induced by TNF-α (25 ng/ml) for 24 h. **(A,B)** Representative western blotting analysis was conducted of protein expression in HL7702 cells after treatment. Quantifications were normalized to β-actin and expressed as relative density. **(C,D)** Representative apoptosis images were showed by TUNEL assay. The number of apoptotic cells was counted using ImageJ software. **(E,F)** A GFP-LC3 plasmid was transfected into cells and the representative fluorescence image of cells was showed. The green spots of cells were manually quantified in five randomly selected areas. **(G,H)** Representative apoptosis images were presented with PE and 7-AAD reagents by flow cytometry. The apoptosis rate was calculated using the proportion of early and late apoptotic cells in total cells.

Tunicamycin (TM) is classified as ERS inducer and 4-phenylbutanoic acid (4-PBA) represents a variety of ERS inhibitor (Park et al., 2018; Wu et al., 2018), both of which can be used to explore the role of ERS in ASPP2 mediated-autophagy and apoptosis. It was discovered that TM treatment increased the expression of GRP78 and Chop, indicating the effectiveness of TM in inducing ERS (Figures 4A,B). Induced by TM, ERS inhibited autophagy in both ASPP2 treated and non-ASPP2 treated groups (Figures 4E,F). Differently, the TM-induced ERS promoted apoptosis in both ASPP2 treated and non-ASPP2 treated groups (Figures 4C,D). However, TM treatment made no difference to the expression of ASPP2, phosphorylated S6 (PS6), phosphorylated mTOR (PmTOR), suggesting a possibility that the regulation of ERS on apoptosis and autophagy could occur in the downstream of ASPP2 and mTOR (Figures 4A,B). In addition, ASPP2 shRNA treatment promoted autophagy, but this effect can be reversed by TM treatment, which implies that ASPP2 inhibited autophagy through ERS pathway (Figures 4E,F). Similarly, ASPP2 shRNA treatment suppressed apoptosis, but this effect can also be reversed by TM treatment, suggesting that ASPP2 promoted apoptosis through ERS pathway (Figures 4G,H). Moreover, 4-PBA treatment verified this results from the opposite perspective (Figure 5). In summary, ERS participated in the regulation of ASPP2 on autophagy and apoptosis while ERS existed in the downstream of ASPP2 and mTORC1.

### ASPP2 Inhibits ERS-Autophagy and Promotes ERS-Apoptosis Through mTORC1 Pathway in Hepatocyte Injury Induce by TNF-α

**FIGURE 6:**
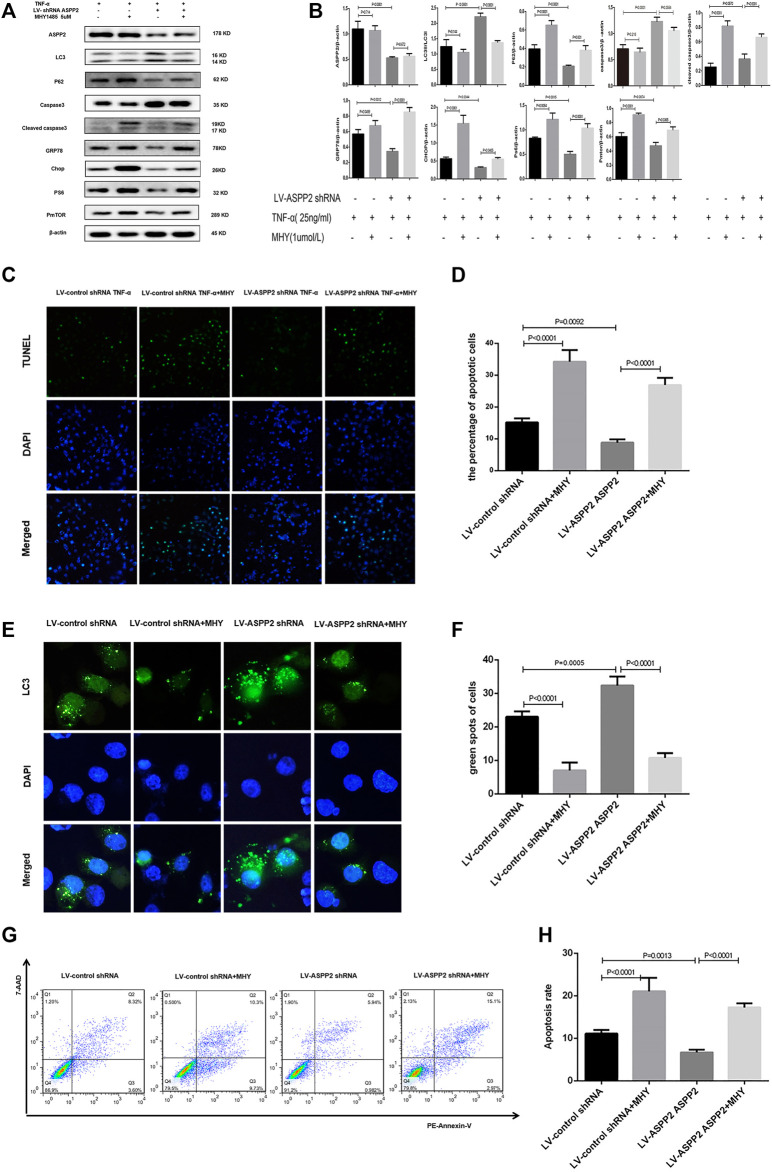
ASPP2 inhibited autophagy but promoted ERS-apoptosis through mTORC1 pathway in the hepatocyte injury induce by TNF-α. HL7702 cells were integrated into stable cell lines using LV-ASPP2shRNA or LV-control shRNA. The stable cell lines were treated with MHYI485 (5 umo/L) and then induced by TNF-α (25 ng/ml) for 24 h. **(A,B)** Representative western blotting analysis was conducted of protein expression in HL7702 cells after treatment. Quantifications were normalized to β-actin and expressed as relative density. **(C,D)** Representative apoptosis images were presented by TUNEL assay. The number of apoptotic cells were calculated using ImageJ software. **(E,F)** A GFP-LC3 plasmid was transfected into cells and the representative fluorescence image of cells were showed. The green spots of cells were manually quantified in five randomly selected areas. **(G,H)** Representative apoptosis images were showed with PE and 7-AAD reagents by flow cytometry. The apoptosis rate was calculated using the proportion of early and late apoptotic cells in total cells.

**FIGURE 7:**
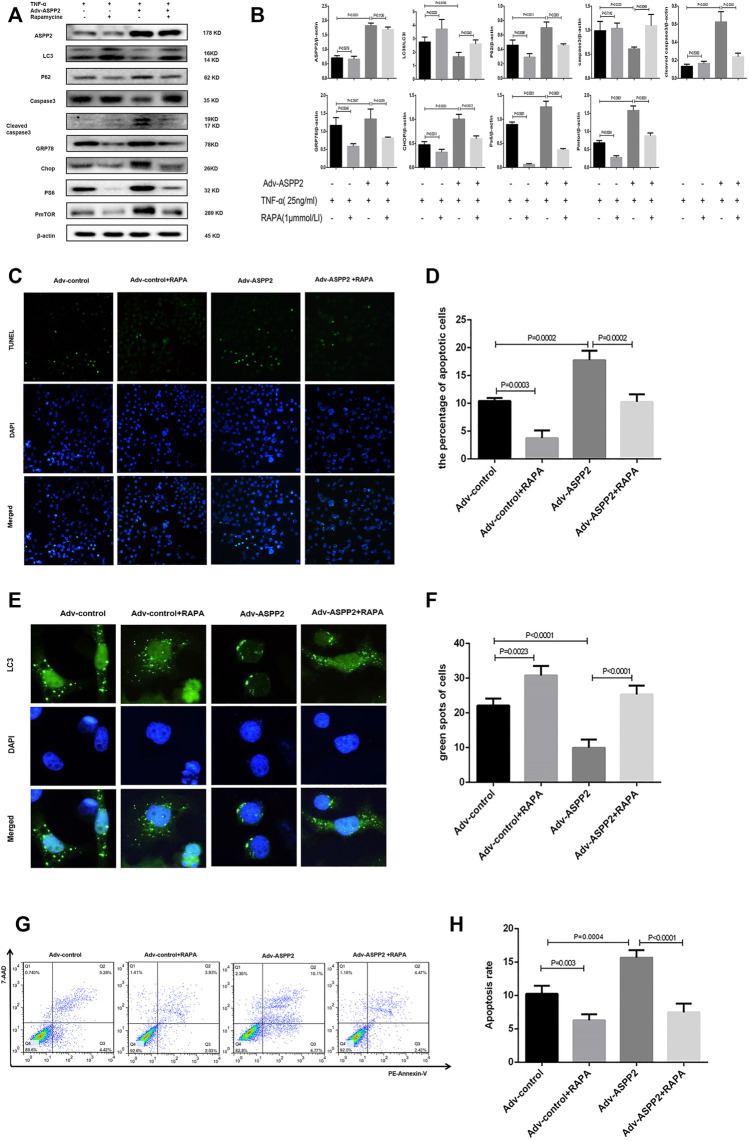
ASPP2 inhibition promoted autophagy but suppressed ERS-apoptosis through mTORC1 pathway in hepatocyte injury induce by TNF-α. HL7702 cells were transfected by Adv-ASPP2 or Adv-control (5 nM), then pretreated by rapamycin (1 μmmol/L) for 2 h and finally induced by TNF-α (25 ng/ml) for 24 h. **(A,B)** Representative western blotting analysis was conducted of protein expression in HL7702 cells after treatment. Quantifications were normalized to β-actin and expressed as relative density. **(C,D)** Representative apoptosis images were presented by TUNEL assay. The number of apoptotic cells was calculated using ImageJ software. **(E,F)** A GFP-LC3 plasmid was transfected into cells and the representative fluorescence image of cells was showed. The green spots of cells were manually quantified in five randomly selected areas. **(G,H)** Representative apoptosis images were showed with PE and 7-AAD reagents by flow cytometry. The apoptosis rate was calculated using the proportion of early and late apoptotic cells in total cells.

MHY1485 is referred to as an effective, cell permeable mTOR agonist, while rapamycin is known as a potent and specific mTOR inhibitor (Benjamin et al., 2011; Lin et al., 2019), both of which can be used to explore the role of mTORC1 in ASPP2 mediated-autophagy and apoptosis. It was found out that ASPP2 shRNA treatment promoted autophagy, but this effect can be reversed by MHY1485 treatment, which suggests that ASPP2 inhibited autophagy through mTORC1 pathway (Figures 6E,F). Similarly, ASPP2 shRNA treatment suppressed apoptosis, but this effect can be reversed by MHY1485 treatment, implying that ASPP2 promoted apoptosis through mTORC1 pathway (Figures 6C,D). MHY1485 treatment increased the expression of PS6 and PmTOR, which means that MHY1485 can activate mTORC1 pathway (Figures 6A,B). MHY1485 treatment reduced autophagy flux in both ASPP2 treated and non-ASPP2 treated groups (Figures 6E,F). Differently, MHY1485 treatment increased apoptosis in both ASPP2 treated and non-ASPP2 treated groups (Figures 6G,H). Despite no effect on the expression of ASPP2, MHY1485 treatment contributed to a significant increase in the expression of GRP78 and Chop, which suggests that the regulation of mTORC1 on apoptosis and autophagy occurred in the downstream of ASPP2 and the upstream of ERS (Figures 6A,B). In addition, rapamycine treatment verified this results from the opposite perspective (Figure 7). In summary, ASPP2 regulated ERS-mediated autophagy and apoptosis through mTORC1 pathway in case of hepatocyte injury induced by TNF-α.

## Discussion

**FIGURE 8:**
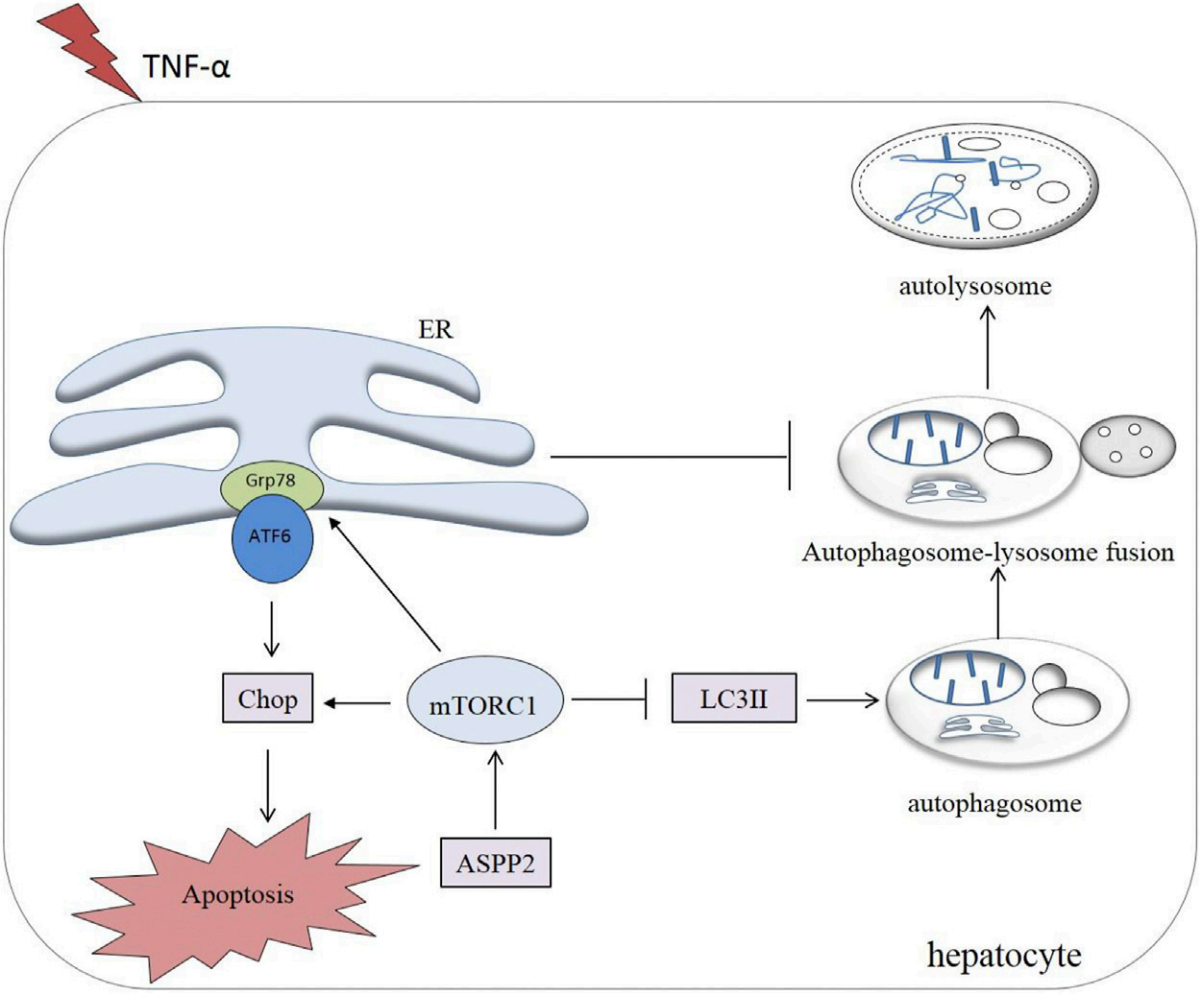
A working model illustrating the role of ASPP2 in the cross-talk between ERS-apoptosis and autophagy. ASPP2 was activated in the hepatocyte injury induced by TNF-α. ASPP2 upregulated mTORC1, inhibited autophagy, and promoted ERS-apoptosis through Grp78 and Chop. mTORC1, downsteam of ASPP2, switched autophagy to ERS-apoptosis of hepatocyte in response to TNF-α. ERS also inhibited autophagy by suppressing the autophagosome-lysosome fusion.

Most importantly, it was discovered in this study that ASPP2 could inhibit autophagy and induce apoptosis through mTORC1-ERS pathway in case of hepatocyte injury induce by TNF-α. Notably, the role of ASPP2-mTORC1-ERS axis was verified in hepatocyte injury, which points to the possibility that ASPP2 can act as an important regulatory molecule in hepatocyte survival and death (Figure 8).

Autophagy is defined as a degradation process dependent on the lysosome commonly found in eukaryotic cells. That is to say, those damaged or redundant proteins and organelles can be encapsulated by double membrane vesicles to generate autophagosome. On this basis, autophagosome is fused with lysosome to generate the autolysosome required to degrade the encapsulated substances (Rautou et al., 2010). The complete process ranging from the formation of autophagosome to the degradation of autolysosome is referred to as autophagic flux (Chun and Kim, 2018). The conversion of microtubule-associated proteins light chain 3 (LC3) I into LC3 II and the degradation of p62 are regarded as the marker of autophagic flux (Yoshii and Mizushima, 2017; Ye et al., 2019). The function of autophagy can be beneficial as well as detrimental, and the severity of autophagy is a determining factor in cell survival and death. In case of mild injury or at the early stage, autophagy is activated for cells to survive by clearing damaged proteins and organelles. Differently, when severe injury occurs or the late stage is reached, autophagy is inhibited and cells enter into the stage of apoptosis or death, thus causing disease (Ueno and Komatsu, 2017; Boutouja et al., 2019). On this basis, it was also found out that autophagy was activated and apoptosis was suppressed to a low level given a low concentration of TNF-α, while autophagy was reduced and apoptosis was significantly increased in high concentration of TNF-α.

So far, there have been an increasing number of studies demonstrating that ASPP2 could play an important role in autophagy and apoptosis. As revealed by Wang et al., ASPP2 can inhibit RAS-induced autophagy by competing with ATG16 to bind ATG5/ATG12 complex and preventing the formation of ATG16/ATG5/ATG12 complex (Wang et al., 2012). Liu et al. discovered that ASPP2 induced the apoptosis of hepatoma cells by dissociating Beclin-1 from Bcl-2-Beclin-1 complex, maintaining the generation of ASPP2-Bcl-2 complex and triggering autophagic apoptosis (Liu et al., 2014). In our previous study, it was revealed that ASPP2 induced liver inflammation and injury by suppressing autophagy and promoting apoptosis (Xu et al., 2019). However, there is still no in-depth investigation conducted into the mechanism that ASPP2 regulates autophagy and apoptosis, especially the key molecular mechanism of interconversion between autophagy and apoptosis.

Previously, it was found out in our study that ASPP2 can regulate mTORC1 pathway (Shi et al., 2018). Yang et al. found out that mTORC1 could drive a shift from autophagy to the apoptosis induced by endoplasmic reticulum stress (ERS) in the chondrocytes in response to the prolonged aberrant biomechanical loadings by upregulating p-EIF2AK3, which can promote the expression of CASP12 and DDIT3. This leads to apoptosis and suppresses autophagy by suppressing autophagosome-lysosome fusion (Yang et al., 2020). In this study, it is suggested that mTORC1 can act as a switch between autophagy and ERS-apoptosis, which plays an important role in cell survival and death. Therefore, it is presumed that ASPP2 has a potential to regulate autophagy and apoptosis through mTORC1. To evidence our hypothesis, it was discovered that ASPP2 regulated ERS-mediated autophagy and apoptosis through mTORC1 pathway in case of hepatocyte injury induced by TNF-α. Especially, it was found out that mTORC1 and ERS participated in the regulation of ASPP2 on autophagy and apoptosis, with mTORC1 and ERS as the regulation molecules in the downstream of ASPP2.

These findings suggest a new regulatory mechanism for hepatocyte apoptosis in the context of liver injury, especially the new molecular mechanism of ASPP2 regulating autophagy and apoptosis. In the future, our further research will be focused on verifying the effect of ASPP2-mTORC1-ERS axis on hepatocyte apoptosis and autophagy through animal experiments, which would contribute to identifying a new target for the treatment of liver injury and liver failure.

## Data Availability

The raw data supporting the conclusions of this article will be made available by the authors, without undue reservation.

## References

[B1] Benjamin D. Colombi M. Moroni C. Hall M. N. (2011). Rapamycin Passes the Torch: a New Generation of mTOR Inhibitors. Nat. Rev. Drug Discov. 10 (11), 868–880. 10.1038/nrd3531 22037041

[B2] Boutouja F. Stiehm C. M. Platta H. W. (2019). mTOR: A Cellular Regulator Interface in Health and Disease. Cells 8 (1), 18. 10.3390/cells8010018 PMC6356367 30609721

[B3] Chun Y. Kim J. (2018). Autophagy: An Essential Degradation Program for Cellular Homeostasis and Life. Cells 7 (12), 278. 10.3390/cells7120278 PMC6315530 30572663

[B4] Hua H. Kong Q. Zhang H. Wang J. Luo T. Jiang Y. (2019). Targeting mTOR for Cancer Therapy. J. Hematol. Oncol. 12 (1), 71. 10.1186/s13045-019-0754-1 31277692 PMC6612215

[B5] Li Y. Ahmad A. Sarkar F. H. (2015). ASPP and iASPP: Implication in Cancer Development and Progression. Cel Mol Biol (Noisy-le-grand) 61 (6), 2–8. 26518890

[B6] Lin X. Peng Z. Wang X. Zou J. Chen D. Chen Z. (2019). Targeting Autophagy Potentiates Antitumor Activity of Met-TKIs against Met-Amplified Gastric Cancer. Cell Death Dis 10 (2), 139. 10.1038/s41419-019-1314-x 30760701 PMC6374362

[B7] Liu K. Shi Y. Guo X. Wang S. Ouyang Y. Hao M. (2014). CHOP Mediates ASPP2-Induced Autophagic Apoptosis in Hepatoma Cells by Releasing Beclin-1 from Bcl-2 and Inducing Nuclear Translocation of Bcl-2. Cel Death Dis 5 (7), e1323. 10.1038/cddis.2014.276 PMC4123070 25032846

[B8] Odle R. I. Walker S. A. Oxley D. Kidger A. M. Balmanno K. Gilley R. (2020). An mTORC1-To-CDK1 Switch Maintains Autophagy Suppression during Mitosis. Mol. Cel 77 (2), 228–e7. 10.1016/j.molcel.2019.10.016 PMC6964153 31733992

[B9] Osna N. A. Donohue T. M. Jr. Kharbanda K. K. (2017). Alcoholic Liver Disease: Pathogenesis and Current Management. Alcohol. Res. 38 (2), 147–161. 28988570 10.35946/arcr.v38.2.01 PMC5513682

[B10] Park H. J. Son H. J. Sul O. J. Suh J. H. Choi H. S. (2018). 4-Phenylbutyric Acid Protects against Lipopolysaccharide-Induced Bone Loss by Modulating Autophagy in Osteoclasts. Biochem. Pharmacol. 151, 9–17. 10.1016/j.bcp.2018.02.019 29458048

[B11] Rautou P. E. Mansouri A. Lebrec D. Durand F. Valla D. Moreau R. (2010). *Autophagy In* Liver Diseases. J. Hepatol. 53 (6), 1123–1134. 10.1016/j.jhep.2010.07.006 20810185

[B12] Rindom E. Kristensen A. M. Overgaard K. Vissing K. de Paoli F. V. (2020). Estimation of p70S6K Thr389 and 4E-BP1 Thr37/46 Phosphorylation Support Dependency of Tension Per Se in a Dose-Response Relationship for Downstream mTORC1 Signalling. Acta Physiol. (Oxf) 229 (1), e13426. 10.1111/apha.13426 31804016

[B13] Seitz H. K. Bataller R. Cortez-Pinto H. Gao B. Gual A. Lackner C. (2018). Alcoholic Liver Disease. Nat. Rev. Dis. Primers 4 (1), 16. 10.1038/s41572-018-0014-7 30115921

[B14] Shi H. Zhang Y. Ji J. Xu P. Shi H. Yue X. (2018). Deficiency of Apoptosis-Stimulating Protein Two of P53 Promotes Liver Regeneration in Mice by Activating Mammalian Target of Rapamycin. Sci. Rep. 8 (1), 17927. 10.1038/s41598-018-36208-3 30560875 PMC6298958

[B15] Turnquist C. Wang Y. Severson D. T. Zhong S. Sun B. Ma J. (2014). STAT1-induced ASPP2 Transcription Identifies a Link between Neuroinflammation, Cell Polarity, and Tumor Suppression. Proc. Natl. Acad. Sci. U S A. 111 (27), 9834–9839. 10.1073/pnas.1407898111 24958857 PMC4103354

[B16] Ueno T. Komatsu M. (2017). Autophagy in the Liver: Functions in Health and Disease. Nat. Rev. Gastroenterol. Hepatol. 14 (3), 170–184. 10.1038/nrgastro.2016.185 28053338

[B17] Vives V. Slee E. A. Lu X. (2006). ASPP2: a Gene that Controls Life and Death *In Vivo* . Cell Cycle 5 (19), 2187–2190. 10.4161/cc.5.19.3266 16969108

[B18] Wang Y. Wang X. D. Lapi E. Sullivan A. Jia W. He Y. W. (2012). Autophagic Activity Dictates the Cellular Response to Oncogenic RAS. Proc. Natl. Acad. Sci. U S A. 109 (33), 13325–13330. 10.1073/pnas.1120193109 22847423 PMC3421174

[B19] Wu J. Chen S. Liu H. Zhang Z. Ni Z. Chen J. (2018). Tunicamycin Specifically Aggravates ER Stress and Overcomes Chemoresistance in Multidrug-Resistant Gastric Cancer Cells by Inhibiting N-Glycosylation. J. Exp. Clin. Cancer Res. 37 (1), 272. 10.1186/s13046-018-0935-8 30413206 PMC6230241

[B20] Xu P. Yao J. Ji J. Shi H. Jiao Y. Hao S. (2019). Deficiency of Apoptosis-Stimulating Protein 2 of P53 Protects Mice from Acute Hepatic Injury Induced by CCl4 via Autophagy. Toxicol. Lett. 316, 85–93. 10.1016/j.toxlet.2019.09.006 31513885

[B21] Yang H. Wen Y. Zhang M. Liu Q. Zhang H. Zhang J. (2020). MTORC1 Coordinates the Autophagy and Apoptosis Signaling in Articular Chondrocytes in Osteoarthritic Temporomandibular Joint. Autophagy 16 (2), 271–288. 10.1080/15548627.2019.1606647 31007149 PMC6984599

[B22] Ye B. Wang Q. Hu H. Shen Y. Fan C. Chen P. (2019). Restoring Autophagic Flux Attenuates Cochlear Spiral Ganglion Neuron Degeneration by Promoting TFEB Nuclear Translocation via Inhibiting MTOR. Autophagy 15 (6), 998–1016. 10.1080/15548627.2019.1569926 30706760 PMC6526833

[B23] Yoshii S. R. Mizushima N. (2017). Monitoring and Measuring Autophagy. Int. J. Mol. Sci. 18 (9), 865. 10.3390/ijms18091865 PMC5618514 28846632

